# Evaluation of a comprehensive intervention with a behavioural modification strategy for childhood obesity prevention: a nonrandomized cluster controlled trial

**DOI:** 10.1186/s12889-015-2535-2

**Published:** 2015-12-03

**Authors:** Jing-jing Wang, Wing-chung Patrick Lau, Hai-jun Wang, Jun Ma

**Affiliations:** Institute of Child and Adolescent Health, School of Public Health, Peking University, Beijing, 100191 China; Department of Physical Education, Faculty of Social Sciences, Hong Kong Baptist University, Hong Kong, China

**Keywords:** Children, Obesity, Comprehensive intervention, Behavioural modification

## Abstract

**Background:**

With regard to the global childhood obesity epidemic, it is imperative that effective lifestyle interventions are devised to combat childhood obesity. This paper describes the development and implementation of a comprehensive (a combination of diet and physical activity (PA)), social cognitive behaviour modification intervention using accelerometry and a dietary diary to tackle child overweight and obesity. The comprehensive intervention effect was evaluated in a comparison with diet only, PA only and a no-treatment control group.

**Methods:**

A pilot study was conducted with a non-randomized cluster design. Four hundred thirty-eight overweight and obese children aged 7–12 years from ten primary schools in Beijing were recruited to receive a one-year intervention. Participants were allocated into one of four groups: the comprehensive intervention group; the PA only group (Happy 10 program); the diet only group (nutrition education program); and a control group. The effects of intervention on adiposity, blood pressure, and biochemical indicators were assessed by examining 2-way interactions (time × intervention) in linear mixed models. Means and 95 % confidence intervals (CI) for the adjusted changes between post-intervention and baseline relative to changes in the control group were calculated and reported as effect sizes.

**Results:**

The percentage of body fat in the comprehensive intervention group showed a significant relative decrease (adjusted change: −1.01 %, 95 % CI: (−1.81, −0.20) %) compared with the PA only, diet only or control groups (*P* < 0.001). Systolic blood pressure significantly decreased in the comprehensive intervention group (adjusted change: −4.37 mmHg, 95 % CI: (−8.42, −0.33) mmHg), as did diastolic blood pressure (adjusted change: −5.50 mmHg, 95 % CI (−8.81, −2.19) mmHg) (*P* < 0.05). Compared with the other two intervention groups and the control group, positive adjusted changes in fasting glucose in the comprehensive group were found, although not for the biochemical lipid metabolism indicators. Positive but non-significant adjusted changes in body mass index and waist circumference were observed.

**Conclusions:**

Compared with the diet or PA only intervention groups, the current comprehensive program had superior positive effects on body fat percentage and blood pressure but not on the biochemical lipid metabolism indicators in Chinese overweight and obese children. Future randomized controlled trials and long-term follow-up studies are required to elaborate the findings of the current intervention.

**Trial registration:**

ClinicalTrials.gov identifier: NCT02228434

**Electronic supplementary material:**

The online version of this article (doi:10.1186/s12889-015-2535-2) contains supplementary material, which is available to authorized users.

## Background

Obesity is a global epidemic and the incidence of childhood obesity has risen dramatically over the past three decades in both developed and developing countries [1]. The standardized prevalence of obesity in Chinese children has increased rapidly from 0.2 % in 1985 to 8.1 % in 2010 [2]. In 2010, the childhood overweight rates in well-developed urban regions in China reached 32.6 % for boys and 19.1 % for girls [3]. Overweight children have been found to be 4.5 times and 2.4 times more likely than normal or underweight children to have elevated systolic blood pressure (SBP) and diastolic blood pressure (DBP), respectively [4]. These children are more likely to have increased risks for heart disease and various chronic diseases later in life, such as hyperlipidaemia, hyperinsulinaemia, hypertension, and type 2 diabetes [5, 6].

Energy dense diets and a lack of physical activity (PA) are the dominant determinants of childhood obesity [7]. Modern lifestyles (which include passive overeating, inactivity and various sociocultural economic influences) in an obesogenic environment have caused the child obesity epidemic to evolve [8]. As such, it is imperative that effective lifestyle interventions be devised and implemented to combat this issue. For overweight and obese children and adolescents, specific strategies are required because these children are more likely to have unhealthy dietary habits [9] and are less inclined to participate in PA initiatives [10]. During the implementation of interventions, nutrition education or PA interventions are considered crucial for preventing weight gain. However, programs including either diet alone or PA alone have shown limited effectiveness for weight loss; thus, it is suggested that interventions acting on both energy intake and energy output are more likely to be effective than those targeting only one aspect [11].

Perceived personal relevance, individualized treatment, engaging information and interactivity are identified as important characteristics of effective interventions that target health behaviour change [12]. These features are prerequisites for moving on to further stages of behavioural change [13]. One major concern is the correct approach to creating and delivering relevant information in a novel and interactive way so the information has the greatest persuasive power. Numerous lifestyle interventions have been implemented to tackle the rate of childhood overweight and obesity; however, researchers have highlighted limitations in several population-based studies, in which causal links between the environment, motivation, interactivity and weight status have not been documented [14]. Recently, multidisciplinary interventions that include cognitive behavioural strategies to help those aiming to lose weight build appropriate mind-sets that are capable of achieving and maintaining lifestyle changes have been recommended [15, 16]. However, there is a paucity of research examining the combination of PA, diet and cognitive behavioural modification strategies in a high-risk group of Chinese overweight and obese children.

The objectives of the present study were to develop, implement and evaluate a comprehensive intervention with a combination of diet, PA, and cognitive behavioural modification strategies for overweight and obese children. In addition, this study compared the effects of the combined intervention to the effects of PA only and diet only interventions. More specifically, the intervention effectiveness was assessed through group comparisons of the following outcomes: 1) adiposity, 2) blood pressure and 3) plasma biochemical glucose and lipid indicators. We hypothesized that the comprehensive intervention would be more effective in reducing levels of obesity-related indicators than would PA or diet intervention alone.

## Methods

### Research design

The study design was a non-randomized, controlled trial with cluster sampling. We contacted the education management department and attained district school lists. Intervention information was delivered to schools, which had similar teaching scales and socio-economic backgrounds. Ten schools that had been approved to participate were finally recruited into the study. The school was assigned as the unit of allocation into one of three intervention groups or a control group to avoid potential contamination between groups. Considering the feasibility of the human resource requirements for the implementation of the comprehensive intervention, only one school was assigned into this group, with three schools in the PA only intervention group, three in the diet only intervention group, and three in the control group. The three intervention groups received the interventions for the duration of one year. Baseline measurements were taken two weeks prior to the beginning of the intervention and post-intervention measurements were conducted after the completion of the intervention. The study was conducted from May 2009 to July 2011 and was approved by the Peking University Biomedical Ethics Committee.

### Participants

Overweight and obese students, defined according to the body mass index (BMI) cut-points used for screening overweight and obesity in Chinese school age children developed by the Working Group for Obesity in China (WGOC), were recruited [17]. Students with any contraindications such as physical diseases (heart, lung, liver, kidney, other vital organs, endocrine diseases and drug side effects) or psychological illnesses that could prevent them from participating in PA or eating a normal diet were excluded. Written informed consent statements were obtained from each participant and their parents before data collection. A total of 438 overweight and obese students aged 7 ~ 12 years (158 males) were enrolled in the baseline tests.

A power analysis was conducted based on the changes in BMI. Power calculations showed that a total of 438 children in 10 schools had a power of 79.6 % to detect a difference of 0.25 units between the post-intervention and baseline measurements in the intervention group relative to the control group, with an intraclass correlation coefficient (ICC) of 0.01 [18]. Because the study was a cluster trial, groups rather than individuals were allocated to different interventions. Within-cluster correlation affects the power of a trial because a greater homogeneity of members in the clusters will increase the standard error of the estimate of the treatment effect, ultimately resulting in a loss of power to detect a difference between the intervention and control groups. Therefore, intervention trials must calculate the ICC to determine the required sample size. Since prior ICC data in the Chinese population are lacking, the ICC of BMI (0.01) in a Western physical activity intervention [19] was used as a reference.

### Intervention groups and content

#### Group 1: comprehensive intervention group

The comprehensive intervention is a combination of PA and diet interventions with the integration of components from social cognitive theory [20] that emphasizes behaviour modification strategies for PA and diet (monitoring, goal-setting, obtaining parental support, and rewarding oneself for achieving goals). Medical research postgraduate students implemented the intervention with the help of physical trainer. Children in this group received the intervention in a cyclical pattern. In total, 6 cycles (two months per cycle) were provided, with breaks during festivals and summer holidays. Each cycle of the comprehensive intervention was conducted with different content according to the following procedures:

##### Step one

Monitoring sessions for PA and diet behaviours: To assess PA, participants were required to wear accelerometers (Model: ZhiJi UX-02 YHKYSci-Tech Development Co., Ltd, Beijing, China) for one week, except during sleep or water-related activities (swimming, showering and bathing). The ZhiJi UX-02 accelerometer is a uniaxial waist-worn accelerometer that has previously been used in chronic disease management in Chinese populations [21]. This accelerometer has a piezoelectric sensor that detects dynamic accelerations and converts them into time spent in different PA intensities (light, moderate, and vigorous).

Dietary monitoring was completed in a purpose-designed diary that was developed with reference to a healthy diet [22] and risky dietary factors for children [23] (see diary sample inAdditional file 1). The diary assessed the frequency (times per week) of the intake of 15 items at breakfast, lunch and dinner items, such as eggs, milk, fruit, meat, vegetables, beans, soft drinks, snacks, as well as fried food, fast food, and eating out (e.g., how many times did you have a soft drink and fried food in the last week?). Once completed, this monitoring information was transferred by researchers into the matched online software that was designed exclusively to analyse ZhiJi UX-02 accelerometer and diary information.

##### Step two

Prescription development: Individualized prescriptions were framed in response to the monitoring results and consisted of two parts: 1) normative feedback, which released normative recommendations of PA levels (60 min of moderate-to-vigorous PA daily) [24] and healthy dietary references for children [22, 23]; and 2) progress feedback, which listed the children’s current status and gave personalized guidelines accordingly (see prescription sample in Additional file 2). The guidelines elaborated goal setting towards improving the PA levels and dietary behaviours, including goals regarding the amount of minutes of PA to be added, and the frequency of risky dietary behaviours to be reduced prior to the next assessment. Children were encouraged to gradually increase their PA levels with a 20 % increment to reach the overall recommended levels (participating in daily MVPA for 60 min) and to reduce unhealthy diet behaviours at each intervention cycle. The written PA prescriptions included recommendations for specified types of PA as well as the intensity, frequency, and duration of both aerobic and strength activities.

##### Step three

Executing the prescription: The prescription was delivered to the students in person, and their parents were informed of the content by phone. Parents were asked to encourage the children to modify unhealthy dietary behaviours and insufficient PA levels towards healthier lifestyles. The display screen on the ZhiJi motion sensor gave recipients immediate feedback, which is known to be helpful in encouraging children to participate effectively in PA and comply with their customized prescriptions. A period of one and a half months was allocated for students to improve their behaviours before conducting the subsequent cycle. Prior to the subsequent monitoring session, a gift that cost ¥10 (approximately US$1.5) was awarded to the children who had reached their goals. In addition, two education lectures about health promotion through nutrition and PA modifications were delivered to all of the parents at the school during parent meetings. A sample individualized prescription was also explained to parents to obtain their support for their children’s behavioural modification. Forty-eight (53.3 %) and 65 (72.2 %) parents attended the two lectures, respectively. Education materials and content were delivered to the parents without attendance by the mail and phone.

#### Group 2: PA only intervention group based on the Happy 10 exercise program

In 1999, education and health experts at the Health Promotion Center of the International Life Science Institute, USA, designed the TAKE 10!™ program [25]. This program included various grade-specific and space-appropriate physical activities for the classroom and were integrated into academic curriculums (e.g., math, science, languages, art). Studies have shown that the program contributed to PA and reinforced academic concepts and skills [26, 27]. Based on the principles of the Take 10!™, the Happy 10 program was initiated by the National Institute for Nutrition and Food Safety of the Chinese Center for Disease Control and Prevention (CDC). The Happy 10 program has been implemented and promoted in urban Beijing since 2004 and is a useful strategy for increasing PA among school children [28]. In the current project, diverse activities, such as ‘invisible rope skipping’, ‘imitating animals’, and the ‘squat and multiplication table’, were linked with the core curriculum objectives and were conducted during breaks. All participating schools in this group were encouraged to conduct two Happy 10 sessions per school day. Activity cards were used to illustrate how to perform the activities, and tracking posters and stickers were used in the classroom to follow progress. In addition, health promotion regarding the benefits of PA, and the harm caused by sedentary lifestyles, was delivered to students and parents in two health education lectures.

#### Group 3: diet only intervention group based on nutrition education program

The nutrition intervention was based on health education lectures given by researchers in the classroom and focused on nutrition and health knowledge, including the components of food nutrients, the importance of eating breakfast, the benefits of fruit, vegetables and water intake, and how to choose healthy snacks. The content of the lectures was revised and adapted through several discussions with experts. Lectures were delivered eight times to students and twice to parents. Each lecture lasted a minimum of 40 min. Furthermore, ‘Dietary Pyramid for Chinese people’ posters were displayed on the walls of all participating classrooms. Cartoon style nutrition education handbooks were also developed by the Department of Student Nutrition at the National Institute of Nutrition and Food Safety, Chinese CDC. Cartoon handbooks containing all of this information were distributed to all participants in the nutrition education group to help clarify the concepts presented in the lectures. Meanwhile, two nutrition education lectures were provided to the parents.

The Happy 10 Exercise and Nutrition Education programs were designed as class-based interventions and were delivered to all of the classes. Only overweight and obese students were included in the current study. The control group received no intervention.

### Outcome measures

All tests were conducted when children arrived at school in the morning. Children were required to fast overnight prior to testing and were offered breakfast upon completion of the tests. All instruments were calibrated before use.

#### Height, weight, BMI and Waist circumference (WC)

All measures were conducted by experienced investigators. The outcome assessors were blinded to treatment allocation. The anthropometric measurements were obtained with the children dressed in light-weight clothing and wearing socks. The measurement procedures followed standardized protocol [29]. Height was measured to the nearest 0.1 cm using a calibrated height measure (GMCS-I, Beijing, China). Weight was measured to the nearest 0.1 kg using a dual leveraged scale (RGT-140, Jiangsu, China). BMI was calculated as weight in kilograms divided by the square of height in meters (kg/m^2^). WC was measured at the midpoint between the lowest rib and the iliac crest using non-elastic Myotape (AccuFitness, Colorado, USA) and recorded to the nearest 0.1 cm.

#### Body composition

Body composition was assessed as the percentage body fat (% BF) using standardized single frequency 50 kHz hand-to-foot bioelectrical impedance analysis with the ImpediMed 50 (ImpediMed Pty Ltd., Queensland, Australia). This method has been validated against underwater weighing among Chinese children and adolescents [30]. The measurements were conducted according to standard procedures with a tetrapolar system connected to four electrodes (3 M Center, St. Paul, MN, USA).

#### Blood pressure

Blood pressure was measured in a seated position after a 5-min rest using a mercury-sphygmomanometer (XJ300/40-1, Shanghai, China) and a stethoscope (KD603, Jiangsu, China). The stethoscope was placed over the brachial artery pulse. SBP was defined as the onset of “tapping” Korotkoff sounds (K1), and DBP was defined as the fifth Korotkoff sounds (K5). Two measurements were taken from all participants at 5-min intervals and were recorded to the nearest 2 mmHg.

All of the above measurements were repeated in sets of two. If each pair of assessments exceeded the specified limits (0.1 cm for height, 0.1 kg for weight, 2 cm for WC, 10 % for % BF, and 5 mmHg for blood pressure), a third attempt was made, and the two closest readings were recorded.

#### Biochemical indicators

Three-millilitre fasting blood samples were obtained by skilled nurses from the department of paediatrics in a local hospital. Biochemical measurements were taken following the standard methods of DeYi Diagnostics Institution in Beijing. The total cholesterol (CHO), triacylglycerol (TG), high density lipoprotein cholesterol (HDL-C), low density lipoprotein cholesterol (LDL-C), and fasting blood glucose (FBG) values were recorded.

### Statistical analyses

The data were input with Epidata 3.1 and analyzed with SPSS20.0. All analyses were performed in line with the intention-to-treat principle. Multiple imputations (MI) with the Markov chain Monte Carlo algorithm [31] were used to handle the missing values of post-intervention non-respondents. In this procedure, demographic variables and baseline outcome measures were used as predictors for regression analyses in the imputation models. Regression analyses for each post-intervention outcome were repeated five times to generate five plausible values. The averages of these five estimates were then integrated into a single data set that was used for the effectiveness analyses.

The baseline characteristics among different groups were compared using chi-square tests for categorical variables and with analysis of variance (ANOVA) for continuous variables. We used a linear mixed model to consider cluster random effects. The effects of treatments on adiposity, blood pressure and biochemical indicators were assessed by examining 2-way interactions (time × intervention) with random effects specified in these analyses, which consisted of adjusting errors for clustering at the school level with a random intercept model. Means and 95 % confidence intervals (95 % CI) for the adjusted changes (the adjusted difference among the three intervention conditions between post-intervention and baseline relative to changes in the control group) were compared with Bonferroni tests in the linear mixed models and were reported as effect sizes. Relative decreases compared with the changes in the control group were considered positive changes for all indicators, except a decrease in HDL-C, which was considered negative. Additionally, the per-protocol analyses were performed with the participants who completed the entire study, and the findings were compared with the intention-to-treat analyses. All statistical significance tests were 2-tailed with *α* = 0.05.

## Results

### Participants in the four groups

**Fig. 1:**
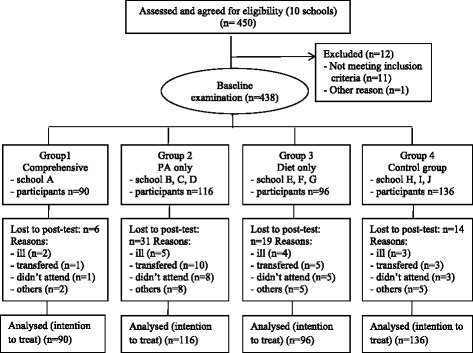
Participants flow diagram

Four hundred thirty-eight students completed the baseline tests (90 in the comprehensive intervention group, 116 in the PA only group, 96 in the diet only group, and 136 in the control group). No significant differences among the four groups were found by age (*P* = 0.319) or gender (*P* = 0.469)(see Table 1). However, there were significant differences in BMI across the four groups (*P* < 0.001). Students in the comprehensive intervention group had higher BMI at baseline. Compared with the other three groups, the intervention group had more obese children (*P* < 0.001). The post-intervention measurements one year later were completed by 368 students (see Fig. 1). The overall dropout rate was 16.0 %, with no gender or age differences between the dropout and retained students (*P* = 0.106). No suspicious outliers were identified for any variable.

### Changes within the comprehensive intervention group

The comprehensive group participants’ dietary behaviours improved between baseline and the final assessment, particularly in the frequencies of breakfast and eating out. The number of times that the children had breakfast increased from 5.6 times per week at baseline to 6.4 times per week post-intervention, and children reduced eating out from 3.2 times per week at baseline to 2.3 times per week after the intervention. At baseline, eighty-five participants provided valid accelerometry-based data. The time children spent in total PA was 293.46 (SD: 76.79) minutes, including 252.08 (SD: 72.34) minutes in light PA and 41.37 (SD: 17.40) minutes in MVPA. After the intervention, children reported higher PA levels, with the total time spent in PA being 315.63 (SD: 81.23) minutes. Time spent in light PA and MVPA were 265.21 (SD: 90.24) and 50.42 (SD: 28.05) minutes, respectively. Nine more children participated in daily MVPA for more than 60 min beyond the ten children who did so at baseline.

### Comparison of effects on adiposity across groups

The results of the intention-to-treat analyses for the anthropometric adiposity measures are presented in Table 2. All participant measures in the current study increased in accordance with the course of the intervention, except for the % BF in the comprehensive intervention group. No significant treatment effects were found in BMI or WC over time among the four groups (both *P* > 0.05). However, of the adjusted changes, post-hoc comparisons showed that students in the comprehensive intervention group had non-significant but greater decreases in BMI (adjusted change: −0.24 kg/m^2^, 95 % CI (−0.73, 0.25) kg/m^2^) and WC (adjusted change: −0.69 cm, 95 % CI (−2.01, 0.62) cm) among the three intervention groups. The adjusted decrease in BMI reached significance compared with the diet only group (adjusted change: 0.36 kg/m^2^, 95 % CI (−0.11, 0.84) kg/m^2^) (*P* = 0.013). A significant treatment effect on % BF over time among the four groups was detected (*P* = 0.025). Compared with the other three groups, the comprehensive intervention group had a significant and greatest relative % BF decrease (adjusted change: −1.01 %, 95 % CI: (−1.81, −0.20) %) (*P* < 0.05).

**Table 1:** Children’s characteristics at baseline

	Comprehensive group (*n* = 90)	PA only group (*n* = 116)	Diet only group (*n* = 96)	Control group (*n* = 136)	*P* value
Gender, *n* (%)					
Boys	35 (38.9)	38 (32.8)	41 (42.7)	44 (32.4)	0.319
Girls	55 (61.1)	78 (67.2)	55 (57.3)	92 (67.6)	
Age (year) mean [SD]	9.41 [1.03]	9.21 [1.28]	9.27 [1.34]	9.16 [1.12]	0.469
BMI (kg/m^2^) mean [SD]	24.48 [3.04]	22.30 [2.83]	22.07 [2.92]	22.25 [2.78]	<0.001
Body weight status, *n* (%)					
Overweight	18 (20.0)	51 (44.0)	55 (57.3)	61 (44.9 %)	<0.001
Obesity	72 (80.0)	65 (56.0)	41 (42.7)	75 (55.1 %)

**Table 2:** The effects of different interventions on Children’s adiposity measures

	Baseline^a^ mean [SD]	Post-intervention^a^ mean [SD]	*P* value	Adjusted change^b^ mean (95 % CI)
Body mass index (BMI, kg/m^2^)				
Comprehensive intervention	24.48 [3.03]	25.28 [3.05]		−0.24 (−0.73, 0.25)^d^
PA only group	22.30 [2.83]	23.33 [3.21]	0.801	−0.01 (−0.47, 0.44)
Diet only group	22.07 [2.92]	23.47 [3.06]		0.36 (−0.11, 0.84)
Control group	22.25 [2.78]	23.29 [2.94]		--
Waist circumference (WC, cm)				
Comprehensive intervention	78.72 [8.50]	81.09 [8.84]		−0.69 (−2.01, 0.62)
PA only group	73.22 [8.69]	76.48 [9.32]	0.947	0.20 (−1.03, 1.42)
Diet only group	73.26 [9.41]	76.67 [9.58]		0.34 (−0.95, 1.63)
Control group	73.71 [8.95]	76.77 [8.47]		--
Percentage of body fat (% BF, %)				
Comprehensive intervention	45.61 [3.38]	45.34 [3.16]		−1.01 (−1.81, −0.20)^c, d, e^
PA only group	41.99 [3.50]	42.65 [3.92]	0.025	−0.07 (−0.82, 0.68)^c^
Diet only group	41.44 [3.72]	43.37 [3.62]		1.20 (0.41, 1.99)^d^
Control group	42.19 [3.51]	42.92 [3.85]		--

^a^Unadjusted mean [SD] were presented for baseline and post-intervention measures

^b^Adjusted change means and 95 % confidence intervals (CIs) are the differences among intervention groups relative to control group by linear mixed model adjusted for baseline value, gender and age

^c^Adjusted differences in groups vs. in diet only group, *P* < 0.05

^d^Adjusted differences in groups vs. in control group, *P* < 0.05

### Comparison of effects on blood pressure across groups

Table 3 provides the results of the intention-to-treat analyses on blood pressure change among the four groups. Reductions in SBP and DBP in the comprehensive group were observed. Significant treatment effects over time in both SBP and DBP were apparent (both *P* < 0.001). Adjusted changes in the control group revealed that the children’s SBP decreased significantly in the comprehensive intervention group (adjusted change: −4.37 mmHg, 95 % CI: (−8.42, −0.33) mmHg) compared with the increases in the PA only and diet only intervention groups (adjusted change in the PA only group: 3.48 mmHg, 95 % CI: (−0.28, 7.27) mmHg; adjusted change in the diet only group: 0.23 mmHg, 95 % CI (−3.74, 4.20) mmHg). Similar to SBP, DBP in the comprehensive intervention group also decreased (adjusted change: −5.50 mmHg, 95 % CI (−8.81, −2.19) mmHg) and was significantly different from the PA only and control groups (*P* < 0.001).

**Table 3:** The effects of different interventions on Children’s blood pressure

	Baseline^a^ mean [SD]	Post-intervention^a^ mean [SD]	*P* value	Adjusted change^b^ mean (95 % CI)
Systolic blood pressure (SBP, mmHg)				
Comprehensive intervention	106.2 [10.3]	103.4 [10.2]		−4.37 (−8.42, −0.33)^c, d, e^
PA only group	107.3 [11.0]	112.4 [10.9]	<0.001	3.48 (−0.28, 7.24)
Diet only group	107.7 [8.7]	109.6 [9.8]		0.23 (−3.74, 4.20)
Control group	104.2 [9.5]	105.8 [9.8]		--
Diastolic blood pressure (DBP, mmHg)				
Comprehensive intervention	66.0 [7.4]	63.1 [6.5]		−5.50 (−8.81, −2.19)^c, e^
PA only group	68.8 [8.4]	69.3 [8.1]	<0.001	−2.15 (−5.23, 0.933)
Diet only group	71.9 [7.5]	69.2 [7.2]		−3.27 (−6.52, −4.02)
Control group	64.2 [7.7]	66.8 [6.2]		--

^a^Unadjusted mean [SD] were presented for baseline and post-intervention measures

^b^Adjusted change means and 95 % confidence intervals (CIs) are the differences among intervention groups relative to control group by linear mixed model adjusted for baseline value, gender and age

^c^Adjusted differences in groups vs. in PA only group, *P* < 0.05

^d^Adjusted differences in groups vs. in diet only group, *P* < 0.05

^e^Adjusted differences in groups vs. in control group, *P* < 0.05

### Comparison of effects on biochemical indicators across groups

The intention-to-treat analyses of biochemical indicators for disease risk (CHO, TG, HDL-C, LDL-C and FBG) are shown in Table 4. Among the children in the schools participating in blood sample collection (except for one control school), blood was not taken from 24 children because they had eaten breakfast on the morning of the testing day. Preliminary health benefitting changes in all of the biochemical indicators were not significant in the comprehensive group except for CHO. There were significant treatment effects over time in CHO, TG, HDL-C, and FBG among the four groups (*P* < 0.05). In the linear mixed model analyses, the greatest relative decreases were only found for FBG in the comprehensive group, and these differences were significant (adjusted change: −0.86 mmol · L^−1^, 95 % CI: (−1.22, −0.50) mmol · L^−1^) compared with the PA only group (adjusted change: −0.54 mmol · L^−1^, 95 % CI: (−0.87, −0.20) mmol · L^−1^) and the diet only group (adjusted change: −0.09 mmol · L^−1^, 95 % CI: (−0.43, 0.26) mmol · L^−1^) (*P* < 0.05), but not in the lipid metabolism indicators.

**Table 4:** The effects of different interventions on Children’s biochemical indicators

	Baseline^a^ mean [SD]	Post-intervention^a^ mean [SD]	*P* value	Adjusted change^b^ mean (95 % CI)
Total cholesterol (CHO, mmol · L^−1^)				
Comprehensive intervention	4.62 [0.89]	4.52 [0.73]		0.49 (0.23, 0.75)^d, e^
PA only group	4.07 [0.65]	3.98 [0.54]	0.004	0.51 (0.26, 0.75)^d, e^
Diet only group	4.12 [0.64]	3.76 [0.51]		0.23 (−0.02, 0.48)^e^
Control group	4.03 [0.65]	3.44 [0.59]		--
Triacylglycerol (TG, mmol · L^−1^)				
Comprehensive intervention	1.24 [0.55]	1.76 [0.87]		0.46 (0.15, 0.78)^c, d, e^
PA only group	1.03 [0.65]	1.01 [0.73]	<0.001	−0.08 (−0.37, 0.21)
Diet only group	1.05 [0.52]	0.85 [0.52]		−0.26 (−0.57, 0.04)
Control group	0.90 [0.48]	0.96 [0.57]		--
High density lipoprotein cholesterol (HDL-C, mmol · L^−1^)				
Comprehensive intervention	1.25 [0.21]	1.05 [0.14]		−0.12 (−0.26, 0.01)^c, d^
PA only group	1.27 [0.27]	1.33 [0.25]	<0.001	0.13 (0.01, 0.25)^d, e^
Diet only group	1.37 [0.32]	1.67 [0.34]		0.38 (0.26, 0.51)^e^
Control group	1.27 [0.23]	1.19 [0.27]		--
Low density lipoprotein cholesterol (LDL-C, mmol · L^−1^)				
Comprehensive intervention	2.67 [0.71]	2.74 [0.71]		0.02 (−0.16, 0.21)
PA only group	1.83 [0.46]	2.04 [0.44]	0.188	0.16 (−0.02, 0.33)^d^
Diet only group	1.86 [0.43]	1.86 [0.45]		−0.06 (−0.24, 0.12)
Control group	1.90 [0.44]	1.95 [0.46]		--
Fasting blood glucose (FBG, mmol · L^−1^)				
Comprehensive intervention	5.45 [0.69]	5.60 [0.65]		−0.86 (−1.22, −0.50)^c, d, e^
PA only group	4.58 [0.42]	5.05 [0.43]	<0.001	−0.54 (−0.87, −0.20)^d, e^
Diet only group	4.28 [0.61]	5.20 [0.39]		−0.09 (−0.43, 0.26)
Control group	4.04 [0.77]	5.05 [0.34]		--

*Note*: Participants number in each group: Comprehensive intervention group: *n* = 73, PA only group: *n* = 115, diet only group, *n* = 92, Control group, *n* = 49

^a^Unadjusted mean [SD] were presented for baseline and post-intervention measures

^b^Adjusted change means and 95 % confidence intervals (CIs) are the differences among intervention groups relative to control group by linear mixed model adjusted for baseline value, gender and age

^c^Adjusted differences in groups vs. in PA only group, *P* < 0.05

^d^Adjusted differences in groups vs. in diet only group, *P* < 0.05

^e^Adjusted differences in groups vs. in control group, *P* < 0.05

In addition, per-protocol analyses were conducted with participants who provided complete data. The results on all outcomes are in line with those reported above from the intention-to-treat analyses (data not shown).

## Discussion

The childhood obesity epidemic is a global burden that has been rapidly increasing in both developed and developing countries. The need for effective intervention strategies has informed the innovative features that have been incorporated into the current comprehensive intervention. The intervention was able to contribute both diet and PA components to the field. One of the novel features of this study is the use of social cognitive theory to support behavioural modification in dietary intake and PA. In the comprehensive intervention, real-time feedback was provided to students regarding their PA and dietary behaviours. Parental support was included in the intervention to facilitate outcomes. The findings of the study provide practical guidelines with which to inform the design of future obesity interventions using wearable PA monitors. Moreover, the current pilot study is considered a valuable contribution to research exploring effective behaviour modification strategies for Chinese childhood obesity. The program prescriptions were scientifically developed and the intervention was carefully designed for overweight and obese children. The effects on adiposity, blood pressure, and biochemical indicators were evaluated and compared against the PA only (with Happy 10 program) and the diet only (with nutrition education program) interventions, which had previously been conducted among Chinese school children in primary school settings [32, 33]. The results demonstrated that the comprehensive intervention resulted in significant improvements in body composition and blood pressure. Positive adjusted FBG changes were detected in the comprehensive intervention compared to the PA only and diet only interventions, but no changes were observed for lipid metabolism. The findings also indicate non-significant but positive adjusted changes in BMI and WC.

As childhood obesity tracks into adulthood [34], more attention should be paid to weight gain during the early years. The comprehensive intervention was designed to achieve goals that were specifically set for each stage using BMI as the primary outcome. In this study, the lack of a comprehensive intervention effect on significant BMI improvement can possibly be attributed to the low intensity of the program. It has previously been reported that other comprehensive interventions with personalized dietary and lifestyle counselling have not managed to modify children’s weight status [35, 36]. Previous interventions delivering counselling letters weekly, bi-weekly or monthly have succeeded in reducing BMI [37, 38]. However, due to the time required for accelerometry-based PA data collection (7 consecutive days in each monitoring cycle), the current study was implemented at a lower frequency with bi-monthly sessions. This may partly account for the positive but non-significant adjusted decrease in BMI in the comprehensive intervention group. A higher intensity intervention program should be considered in the future to enhance exposure to the treatment.

A previous study that adopted two daily Happy 10 sessions demonstrated a treatment effect of lowering children’s fat mass and body composition compared with a control group [32]. However, no improvements in body fat were found in the PA only intervention group with the Happy 10 program in the current study. This discrepancy may be attributed to the different participants in the program. Unlike the previous study that included a large portion of healthy weight children [32], only overweight and obese children were recruited into the present study, and the lack of observed intervention effects suggests the greater need for PA and energy expenditure in overweight and obese children. Although the outcome measures are expected to increase, as part of normal growth and development, it is noteworthy that only students in the comprehensive intervention demonstrated a slight % BF reduction post-intervention. Compared with the control group, the comprehensive intervention showed a favourable adjusted decrease (−1.01 %, 95 % CI (−1.81, −0.20)) in body composition, which was significant compared with the PA only and diet only groups. One study with 96 overweight and obese children, which was based on a lifestyle intervention, achieved a treatment effect on the reduction of % BF (95 % CI = −3.31 to −1.55) [39]. Considering the age-related increase in body fat that would normally occur during early adolescence, the typical effort required to halt the speed of body fat gain suggests that the current findings are noteworthy and are considered a success. Similar to previous studies [40, 41] measuring adiposity parameters, inconsistencies in the current % BF and BMI intervention effects suggest a follow-up test to determine whether similar improvements in BMI can be induced.

The beneficial decreases in blood pressure as a result of the comprehensive intervention are also noteworthy. In the comprehensive intervention, SBP and DBP both dropped significantly post-intervention. SBP had the greatest relative decrease (adjusted change: 4.37 mmHg, 95 % CI (−8.42 to −0.33) mmHg), followed by DBP (adjusted change: 5.50 mmHg, 95 % CI (−8.81 to −2.19) mmHg), across the three intervention groups compared with the adjusted changes observed in the control group; these changes are in line with previous studies by Savoye et al. [38] and Elizondo et al. [39]. In the PA only group, a reduction in SBP and DBP did not occur after the intervention. Mcmurray et al. [40] and other researchers [41–43] have described the significant effects of exercise programs on blood pressure. In contrast, the relationship between PA levels and blood pressure were not found in studies by Guillaume et al. [44] and Klesges et al. [45]. These diverse results could be explained by the fact that blood pressure is correlated with aerobic abilities (VO_2_ max), which are determined by the type and intensity of exercise rather than PA volume alone [46]. The Happy 10 exercise program was designed as a series of classroom-based activities; therefore, the PA intensity in the current study was limited, and its duration was shorter than the recommended level, which may partly explain why this intervention had insufficient power to lower blood pressure. In the diet only group, though DBP decreased after the intervention, no consistent SBP findings exist in the literature. In fact, it has been suggested that education interventions alone are not expected to have much of an effect on young adolescent blood pressure [40, 47]. The success of the comprehensive intervention in the current study may be attributed to the use of monitoring, feedback, problem solving, and reinforcement combined with PA and diet to encourage participants’ motivation.

Moreover, several biochemical disease risk indicators were tested in the current study. It is well known that the prevalence of metabolic syndrome is high among obese children and adolescents and that the magnitude of this risk increases with the severity of obesity. Biochemical indicators with a higher risk of adverse cardiovascular outcomes are present in obese youngsters [48]; however, in the comprehensive intervention group, the current preliminary findings revealed no improvement in most of the plasma biochemical indicators. Considering the comparison of adjusted changes, only a relative positive and significant decrease in FBG was observed in the comprehensive group, but no decrease was found for the lipid metabolism indicators. This could be partly attributed to the fact that there was no reduction in BMI in this study. Previous studies have found that improvements in biochemical indicators are most often accompanied by an observed reduction in BMI, WC, or % BF [38, 49, 50]. Although a slight reduction in % BF was found, significant decreases in BMI and WC were not evident in the comprehensive intervention group. The existing studies suggest that modest, sustained weight loss can have long-term benefits on biochemical disease risk indicators [51, 52]. The effect of interventions on biochemical indicators may be potentially significant, or stronger, if they are concurrent with weight loss [53].

### Strengths and limitations

One of the notable strengths of the present comprehensive intervention is the integration of a social cognitive behavioural program, which is considered an appropriate strategy with which to increase children’s awareness and willingness to participate in more PA and to develop healthier eating habits [54]. Individual prescriptions result in less redundant information, provide focused attention on health outcomes, and encourage engagement and interactivity [55]. A comprehensive intervention with individualized prescriptions is an engaging method of improving children’s adherence to healthier lifestyles [56]. Additionally, parenting is key to developing a home environment that fosters healthy eating and PA among youth [57]. Because children, particularly students in primary school, do not always have full control over their own diet and PA behaviours, one of marked features of the intervention is getting parental support to shape their children’s behaviours, which could facilitate better outcomes. Furthermore, ZhiJi accelerometers were adopted as the monitoring instrument, which is distinct from most previous similar social cognitive behavioural intervention studies that have relied on self-report instruments [58, 59]. Self-report measures for PA have well-documented inherent limitations and tend towards biases because children are less likely to self-report PA accurately [60]. Accelerometry-based motion detectors are wearable objective measures that provide accurate recordings of the intensity and duration of PA. This type of measurement can describe the pattern of children’s PA levels effectively and thus offers strong support of the modification of overall health behaviours. This study compared the comprehensive intervention with PA and nutrition interventions rather than making comparisons solely with a control group. Moreover, the evaluation of the groups’ relative effectiveness using post-hoc tests offers a much more complete picture of the effectiveness of the intervention.

The limitations of this study should be carefully considered. First, the quasi-experimental design, with the school allocated as the unit, resulted in a difference in the proportion of obese children at baseline. The school in the comprehensive intervention included students with significantly higher BMI than the other groups. There is a possibility that participants in this group had a stronger intention to improve than did the children in the other groups. However, the difficulties of dieting and keeping weight off are well documented. Fighting temptation is difficult, and for the obese, these struggles may be even more extreme [61]. Though the baseline values were adjusted in the linear mixed models, a greater risk of bias has resulted from an imbalanced allocation, which might impact the findings across the four groups. Second, this study only explored the effects of the comprehensive intervention compared with two single-sided (PA or diet) interventions. Therefore, a comparison study with other combined PA and diet interventions without the adoption of social cognitive behavioural strategies is required to further examine its effectiveness. Additionally, the study results are from overweight and obese children who volunteered to participate. It is possible that these students are not representative of all children of similar ages; therefore, the results may not be generalizable to all paediatric populations. Finally, it is unclear whether the short-term effect of the intervention on adiposity and blood pressure can be maintained over time because no follow-up tests were conducted.

## Conclusion

In summary, this pilot study has demonstrated the potential of a social cognitive behaviour strategy intervention. The intervention was comprehensive in its approach and generated improvements in children’s body composition and blood pressure. Additionally, positive adjusted changes in BMI, WC and fasting glucose metabolism were demonstrated compared with PA only or diet only interventions but not in the biochemical lipid metabolism indicators. The applications of the intervention strategies are feasible within health promotion programs for overweight and obese children. In the future, the implementation of this intervention among other high-risk populations within randomized clinical trials and long-term follow-up studies is required to further elaborate its potential effectiveness.

## Additional files

Additional file 1: Dietary behaviors diary. (DOC 42 kb) Additional file 2: Individual prescription. (XLS 71 kb)
