# Supplementary material for: Evaluation of a comprehensive intervention with a behavioural modification strategy for childhood obesity prevention: a nonrandomized cluster controlled trial
Source: BMC Public Health. 2015 Dec 3;15:1206. doi: 10.1186/s12889-015-2535-2 (PMC4668691; doi:10.1186/s12889-015-2535-2)
Supplement: Additional file 1: — Dietary behaviors diary. (DOC 42 kb) [file 12889_2015_2535_MOESM1_ESM.doc]

**Dietary behaviors diary**

Do you have the following items today? Please click Yes/No according to your eating.

Date (yyyy/mm/dd) Mon / Tue / Wed / Thur /Fri / Sat / Sun

| Option  Items | Yes/No | Notes |
| --- | --- | --- |
| 1. Breakfast | Yes □  No □ |  |
| 2.Staple foods in lunch (e.g.,noodle, rice,steamed bread) | Yes □  No □ |  |
| 3.Staple foods in dinner (e.g.,noodle, rice,steamed bread) | Yes □  No □ |  |
| 4. Egg | Yes □  No □ |  |
| 5. Milk/Yoghourt | Yes □  No □ | Milk：Bat shape□ bags (200ml/bag)  Box shape□ boxes (250/box)  Yoghourt：Bat shape□ bags (2ml/bag)  Box shape□ boxes (250/box |
| 6. Vegetables | Yes □  No □ |  |
| 7. Fruit | Yes □  No □ |  |
| 8. Meat (e.g.,chiken,duck meat, fish, pork, beef) | Yes □  No □ |  |
| 9. Beans and products | Yes □  No □ |  |
| 10. Soft drink (e.g.,coca-cola, 7-up) | Yes □  No □ | Can amount：1 □ 2 □ 3 □ ≥3 □  Bottle amount：1□ 2 □ 3 □ ≥3 □ |
| 11.Unhealthy snack | Yes □  No □ | Candy（lollipop,Chocolate etc.） □  Puffed food（shrip bar,bugles etc.） □  Sweet Dim Sum（pie, caramel treats etc.） □  French fries, patato chips □  Others |
| 12. Fast food | Yes □  No □ | Mcdonald's □ KFC □ Pizza Hut □  Dicos □ Others |
| 13. Fried foods | Yes □  No □ | Deep-fried dough sticks □  Fried chicken wings etc □  Others |
| 14. Eating-out | Yes □  No □ | Once □ 2-3 times □ ≥ 3 times□ |
| 15. Dinner time: | The interval between dinner and sleep time: hour minutes | |
